# 巴利昔单抗治疗胃肠道慢性移植物抗宿主病的疗效及安全性

**DOI:** 10.3760/cma.j.cn121090-20250630-00306

**Published:** 2026-02

**Authors:** 晓璐 朱, 景枝 王, 海霞 付, 郑丽 徐, 育红 陈, 晓冬 莫, 晓辉 张, 兰平 许, 晓军 黄, 昱 王

**Affiliations:** 北京大学人民医院、北京大学血液病研究所、国家血液系统疾病临床医学研究中心、造血干细胞移植治疗血液病北京市重点实验室，北京 100044 Peking University People's Hospital, Peking University Institute of Hematology, National Clinical Research Center for Hematologic Disease, Beijing Key Laboratory of Hematopoietic Stem Cell Transplantation, Beijing 100044, China

**Keywords:** 慢性移植物抗宿主病, 腹泻, 巴利昔单抗, Chronic graft-versus-host disease, Diarrhea, Basiliximab

## Abstract

**目的:**

初步探索巴利昔单抗治疗胃肠道（GI）慢性移植物抗宿主病（cGVHD）的有效性及安全性。

**方法:**

回顾性病例研究。纳入2018年1月1日至2023年12月31日在北京大学血液病研究所接受移植后发生GI-cGVHD的患者，分析应用巴利昔单抗的有效率、起效时间、总生存（OS）率，以及中性粒细胞减少、贫血、血小板减少和感染的发生率。

**结果:**

纳入41例GI-cGVHD患者，中位随访时间622（112～2 418）d。所有患者初始治疗均包含糖皮质激素、钙调磷酸酶抑制剂和（或）其他免疫抑制剂，如霉酚酸酯、间充质干细胞、西罗莫司等。巴利昔单抗治疗组（25例）患者基线腹泻级别为2～4级，其中2级4例，3级18例，4级3例。该组患者在初始治疗启动后中位时间6 d内启动了巴利昔单抗治疗，其中12例（48.0％）患者接受了4剂以上巴利昔单抗治疗。该组22例（88.0％）患者获得完全缓解。在获得完全缓解的患者中，15例（68.2％）在4剂内（含4剂）实现缓解。非巴利昔单抗治疗组（16例）基线腹泻级别较巴利昔单抗治疗组低（*P*<0.001），均为1～2级，其中1级12例，2级4例。所有患者均获得完全缓解。两组治疗有效率差异无统计学意义（88.0％对100.0％，*P*＝0.150）。巴利昔单抗治疗组和非巴利昔单抗治疗组2年OS率分别为75.4％和86.7％（*P*＝0.494），2年累积复发率分别为16.9％和14.3％（*P*＝0.913），2年非复发死亡率分别为14.2％和6.7％（*P*＝0.526）。巴利昔单抗治疗组未观察到新发的3～4级肝肾功能异常、细胞因子释放综合征。该组中性粒细胞减少、贫血和血小板减少的发生率分别为24.0％、28.0％和28.0％。任何感染、病毒感染、细菌感染和真菌感染的发生率分别为52.0％、36.0％、32.0％和4.0％。

**结论:**

巴利昔单抗可用于治疗GI-cGVHD，且其血液学不良反应和感染性不良事件发生率与其他全身性免疫抑制剂相当。该研究结果为GI-cGVHD患者提供了潜在治疗选择，但仍需进一步前瞻性大样本临床研究验证其安全性。

## Abstract (en)

**Objective:**

To explore the efficacy and safety of basiliximab for gastrointestinal（GI）chronic graft-versus-host disease（cGVHD）.

**Methods:**

This was a retrospective case series. Patients who developed GI-cGVHD after transplantation at the Peking University Institute of Hematology between January 1, 2018, and December 31, 2023, were included. Outcomes included response rate, time to response, overall survival（OS）, and the incidence of neutropenia, anemia, thrombocytopenia, and infections.

**Results:**

A total of 41 patients with GI-cGVHD were included, with a median follow-up of 622（range, 112–2 418）days. All patients received initial therapy including glucocorticoids, calcineurin inhibitors, and/or other immunosuppressive agents（eg, mycophenolate mofetil, mesenchymal stem cells, and sirolimus）. In the basiliximab group（*n*＝25）, baseline diarrhea was grade 2–4（grade 2, *n*＝4; grade 3, *n*＝18; grade 4, *n*＝3）. Basiliximab was initiated a median of 6 days after initial therapy, and 12 patients（48.0％）received >4 doses. In the basiliximab group, 22 patients（88.0％）achieved complete response（CR）. Among patients who achieved CR, 15（68.2％）responded within ≤4 doses. In the non-basiliximab group（*n*＝16）, baseline diarrhea was significantly milder than in the basiliximab group（*P*<0.001）and was grade 1–2 in all patients（grade 1, *n*＝12; grade 2, *n*＝4）. All patients in this group achieved CR. The response rate did not differ significantly between groups（88.0％ *vs* 100.0％, *P*＝0.150）. The 2-year OS rates were 75.4％ and 86.7％（*P*＝0.494）, the 2-year cumulative relapse rates were 16.9％ and 14.3％（*P*＝0.913）, and the 2-year non-relapse mortality rates were 14.2％ and 6.7％（*P*＝0.526) in the basiliximab and non-basiliximab groups, respectively. No new grade 3–4 hepatic or renal dysfunction or cytokine release syndrome was observed in the basiliximab group. In the basiliximab group, neutropenia, anemia, and thrombocytopenia occurred in 24.0％, 28.0％, and 28.0％ of patients, respectively. Any infection, viral infection, bacterial infection, and fungal infection occurred in 52.0％, 36.0％, 32.0％, and 4.0％ of patients, respectively.

**Conclusion:**

Basiliximab may be used to treat GI-cGVHD, with hematologic and infection-related adverse event rates comparable to those of other systemic immunosuppressants. These findings suggest a potential therapeutic option for GI-cGVHD; however, larger prospective studies are needed to further evaluate its safety.

慢性移植物抗宿主病（Chronic graft versus host disease，cGVHD）是异基因造血干细胞移植（allo-HSCT）后的严重并发症，30％～70％的长期存活者会受其影响[1]–[2]。胃肠道（Gastrointestinal tract，GI）是cGVHD最常累及的器官，40％～50％的患者会出现胃肠道受累[3]。其临床表现包括腹泻、腹痛、恶心、呕吐及体重下降等，严重影响患者生活质量和生存预后。

目前系统性免疫抑制治疗是GI-cGVHD的主要治疗手段。糖皮质激素（如泼尼松）作为一线用药[4]，长期大剂量使用会带来显著不良反应。钙调磷酸酶抑制剂（如环孢素）常与激素联用，其他免疫抑制剂如甲氨蝶呤[5]、霉酚酸酯[6]、芦可替尼[7]及西罗莫司[8]等疗效各异。由于GI-cGVHD发病机制复杂且现有治疗手段效果有限，其临床管理仍具挑战性。然而，对cGVHD一线治疗疗效进行初步评估至少需要1个月时间[2],[9]，而在此期间患者腹泻量可能已显著升高，甚至出现消化道出血、肠梗阻等严重合并症，这将极大增加后续治疗难度。因此，与治疗严重肝脏cGVHD相似[10]，对严重和（或）预后较差GI-cGVHD患者及时启动二线治疗可能有助于提升疗效。巴利昔单抗能特异性结合活化T细胞表面的IL-2受体α链（CD25），阻断IL-2介导的T细胞活化与增殖[11]。鉴于同种反应性T细胞在cGVHD发病机制中的核心作用[12]，该药物可能对GI-cGVHD具有治疗潜力。我中心前瞻性研究提示巴利昔单抗有助于控制严重肝脏cGVHD[10]，且其血液学不良反应和感染发生率与对照组相当。目前关于巴利昔单抗治疗cGVHD的数据有限。因此，本研究旨在评估巴利昔单抗联合糖皮质激素和钙调磷酸酶抑制剂治疗GI-cGVHD的疗效与安全性。

## 对象与方法

一、研究对象

本研究为回顾性队列研究。选取2018年1月1日至2023年12月31日在北京大学血液病研究所接受allo-HSCT的年龄≥18岁恶性血液病患者。纳入标准：①符合cGVHD诊断。②具有以下临床表现、内镜及病理特征。临床症状包括：持续性恶心呕吐、腹痛腹泻；吸收不良导致的体重下降和低蛋白血症；厌食及进食回避。特征性病理改变包括：肠隐窝凋亡小体增多（每个隐窝≥1个）；隐窝或表面上皮淋巴细胞浸润；隐窝结构破坏及杯状细胞减少。内镜下可见：黏膜水肿充血；黏膜脆性增加、糜烂或溃疡；出血或渗出。影像学（CT/MRI）可显示肠壁增厚水肿。排除标准：①感染性及药物性肠病患者[2]。②肝脏、肺部cGVHD患者。不良事件按CTCAE 5.0标准评估。本研究经北京大学人民医院伦理委员会批准（2020PHB170-01），遵循《赫尔辛基宣言》。

二、方法

1. 预处理方案[13]：HLA亲缘单倍型、非血缘全相合移植应用BU-CY+ATG方案或TBI-CY+ATG预处理方案，具体为：阿糖胞苷（Ara-C）4 g/m^2^，−9 d；白消安（BU）0.8 mg/kg，每6 h 1次，−8 d至−6 d；环磷酰胺（CY）1.8 g/m^2^，−5 d至−4 d；抗胸腺细胞免疫球蛋白（ATG）2.5 mg/kg，−5 d至−2 d；司莫司汀250 mg/m^2^, −3 d。母系或旁系单倍型供者在移植后加用低剂量环磷酰胺（PTCy），14.5 mg·kg^−1^·d^−1^，+3、+5 d。高龄或造血干细胞移植合并症指数（HCT-CI）≥3的患者采取减低毒性预处理（RTC）方案，即BU-FLU/CY+ATG方案，具体为：Ara-C 4 g/m^2^, −9 d；BU 0.8 mg/kg，每6 h 1次，−8 d至−6 d；CY 1.0 g/m^2^，−5 d至−4 d；氟达拉滨30 mg/m^2^，−6 d至−2 d；ATG 2.5 mg/kg，−5 d至−2 d；司莫司汀250 mg/m^2^，−3 d[14]–[16]。

2. GVHD预防方案[13]：采用环孢素+短程甲氨蝶呤+霉酚酸酯方案进行GVHD的预防。

3. 巴利昔单抗给药方案：体重≥35 kg者20 mg/次，<35 kg者10 mg/次，第1、3、8天给药，后续每周重复。该组别患者初始治疗均包含糖皮质激素、钙调磷酸酶抑制剂和（或）其他免疫抑制剂，如霉酚酸酯（5例）、间充质干细胞（6例）、西罗莫司（1例）。

4. 定义：由于GI-cGVHD美国国立卫生研究院（NIH）共识[1]的分级及疗效评估标准未对腹泻量明确规定，本研究参照急性GVHD国际联盟（MAGIC）分级标准和疗效评估标准[17]–[18]：0级：成人<500 ml/d或<3次/d；1级：成人500～999 ml/d或3～4次/d；2级：成人1000～1500 ml/d或5～7次/d；3级：成人>1500 ml/d或>7次/d；4级：严重腹痛伴或不伴肠梗阻或便血（无论排便量如何）。完全缓解（CR）指受累器官的GVHD表现完全消失；部分缓解（PR）指初始受累器官的GVHD改善（至少降低一个级别）但未达到CR；无反应（NR）指受累器官的GVHD严重程度无改善也没有恶化或患者死亡；进展（PD）指靶器官的GVHD加重（至少增加一个级别）。PD和NR为治疗无效[17]。非复发死亡是指与疾病复发/进展无关的死亡。感染诊断标准参照文献[19]–[22]标准。

5. 随访：采用查阅门诊/住院病历及电话联系等方式进行随访。随访截止时间为2025年1月31日。中位随访时间622（112～2 418）d。

三、统计学处理

数据分析采用SPSS25.0统计软件进行。连续变量以中位数（范围）表示，分类变量以百分比表示。分类变量组间比较采用*χ*^2^检验。总生存（OS）时间定义为从供者干细胞回输后第1天至随访结束或因任何原因死亡的时间。OS采用Kaplan-Meier曲线进行分析，采用Log-rank检验进行比较。采用Fine-Gray竞争风险模型，移植相关死亡的竞争风险为复发。所有统计中*P*<0.05（双侧）表示差异有统计学意义。

## 结果

1. 患者临床特征：本研究筛选了63例在我中心接受allo-HSCT后，出现cGVHD伴GI受累的成人血液恶性疾病患者，除外接受供者淋巴细胞回输2个月内新发GI受累患者，最终共纳入41例GI-cGVHD患者，8例因原发病分子学复发接受了干扰素治疗，余33例处于CR状态。41例患者中32例合并皮肤cGVHD（26例NIH评分1分，6例评分2分）；21例合并眼部cGVHD（19例评分1分，2例评分2分）；18例合并口腔cGVHD（15例评分1分，3例评分2分）；2例合并关节和筋膜cGVHD（均评分1分）。患者入组距离HSCT中位时间201（114～577）d，中位随访时间622（112～2 418）d。巴利昔单抗治疗组（25例）患者中位年龄为45岁，原发病包括急性髓系白血病（AML）、急性淋巴细胞白血病（ALL）、骨髓增生异常肿瘤（MDS）。该组中22例接受了亲缘单倍型相合HSCT，另3例接受了非血缘全相合HSCT。所有患者基线腹泻级别均为2～4级，其中2级4例，3级18例，4级3例。该组13例为cGVHD预后危险度分组（Risk group, RG）3组（7～9分），2例为RG 4组（≥10分）。非巴利昔单抗治疗组（16例）患者中位年龄为38岁，该组中15例接受了亲缘单倍型相合HSCT，另1例接受了非血缘全相合HSCT。该组患者基线腹泻级别较巴利昔单抗治疗组低（*P*<0.001），均为1～2级，其中1级12例，2级4例。患者基线临床特征见表1。

**表1:** 41例胃肠道慢性移植物抗宿主病患者临床特征

特征	巴利昔单抗治疗组（25例）	非巴利昔单抗治疗组（16例）	*P*值
年龄［岁，*M*（范围）］	45（19～61）	38（18～61）	0.342
性别（例，男/女）	12/13	9/7	0.606
原发病［例（％）］			0.576
AML	14（56.0）	11（68.8）	
ALL	10（40.0）	5（31.2）	
MDS	1（4.0）	0（0）	
供者类型［例（％）］			0.545
亲缘单倍型	22（88.0）	15（93.8）	
非血缘全相合	3（12.0）	1（6.2）	
ABO血型［例（％）］			0.444
相合	11（44.0）	9（56.3）	
不相合	14（56.0）	7（43.7）	
基线腹泻级别［例（％）］			<0.001
1级	0（0）	12（75.0）	
2级	4（16.0）	4（25.0）	
3级	18（72.0）	0（0）	
4级	3（12.0）	0（0）	
其他免疫抑制剂［例（％）］			/
霉酚酸酯	5（20.0）	1（6.3）	
间充质干细胞	6（24.0）	1（6.3）	
西罗莫司	1（4.0）	0（0）	

**注** AML：急性髓系白血病；ALL：急性淋巴细胞白血病；MDS：骨髓增生异常肿瘤；/：不适用

2. 巴利昔单抗治疗GI-cGVHD的有效性：所有患者初始治疗均包含糖皮质激素、钙调磷酸酶抑制剂和（或）其他免疫抑制剂如霉酚酸酯、间充质干细胞、西罗莫司等（表1）。巴利昔单抗治疗组患者在初始治疗启动后中位时间6（1～13）d内启动了巴利昔单抗治疗。启动时间≥6 d患者中，CR率为84.6％（11/13），启动时间<6 d患者中，CR率为91.7％（11/12），差异无统计学意义（*P*＝0.588）。其中10例（40.0％）患者接受了4剂以上（5～7剂）巴利昔单抗治疗，15例（60.0％）患者接受了4剂以下（2～4剂）治疗。

经巴利昔单抗治疗后，22例（88.0％）患者达到CR（图1），中位达缓解时间为24（10～62）d。在随访≥1年的16例患者中，100％患者达到CR。在获得CR的患者中，15例（68.2％）在4剂内（含4剂）实现缓解。启动时间≥6 d患者中，巴利昔单抗使用4剂以上（5～7剂）占比为53.8％（7/13），启动时间<6 d的患者中，巴利昔单抗使用4剂以上占比为41.7％（5/12），差异无统计学意义（*P*＝0.543）。

**图1:**
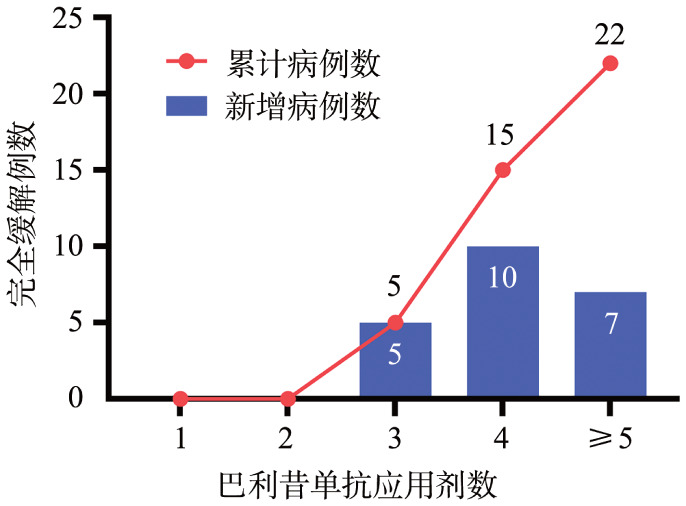
25例巴利昔单抗治疗组患者应用剂数与缓解例数

非巴利昔单抗治疗组，所有患者均获得CR，其中87.5％（14/16）接受一线治疗（激素±钙调磷酸酶抑制剂）获得CR，另2例除一线治疗外，接受了二线治疗，1例加用霉酚酸酯获得CR，1例加用间充质干细胞输注治疗获得CR。中位达缓解时间15（6～54）d。两组治疗有效率差异无统计学意义（*P*＝0.150）。

在随访期内，巴利昔单抗治疗组5例患者死亡，其中3例为非复发死亡（1例为败血症，发生在移植后第309天，入组后第201天，应用巴利昔单抗7剂；1例为中枢神经系统感染，发生在移植后第245天，入组后第112天，应用巴利昔单抗5剂；1例为重症肺炎，发生在移植后第523天，入组后第255天，应用巴利昔单抗6剂），另2例均为复发死亡，分别发生在移植后第405、578天，入组后第177、324天。非巴利昔单抗治疗组2例患者死亡，1例为复发死亡，发生在移植后第352天、入组后第201天，1例为非复发死亡，死于重症肺炎，发生在移植后第301天，入组后第252天。

巴利昔单抗治疗与非巴利昔单抗治疗的患者2年OS率分别为75.4％和86.7％（*P*＝0.494）（图2），2年累积复发率（含血液学复发、分子学复发）分别为16.9％和14.3％（*P*＝0.913），2年非复发死亡率分别为14.2％和6.7％（*P*＝0.526）。

**图2:**
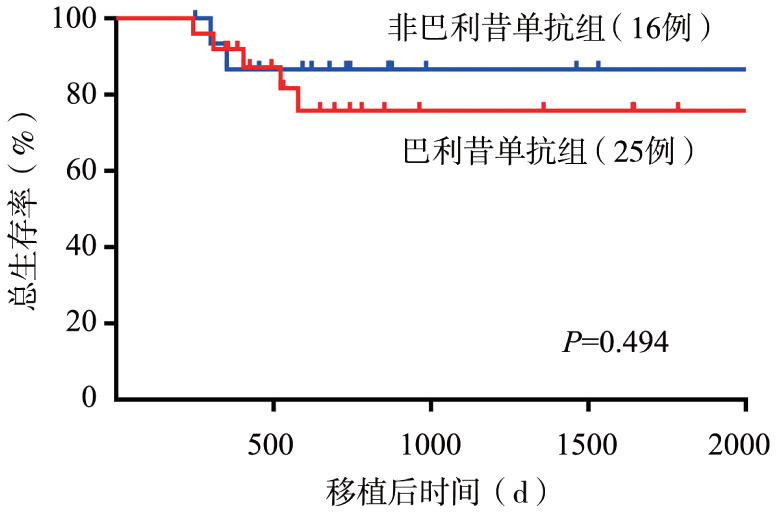
41例胃肠道慢性移植物抗宿主病患者总生存曲线

3. GI-cGVHD治疗的安全性评估：巴利昔单抗治疗期间未观察到明显的输液或过敏反应。该组中性粒细胞减少、贫血和血小板减少的发生率分别为24.0％、28.0％和28.0％。任何感染、病毒感染、细菌感染和真菌感染的发生率分别为52.0％、36.0％、32.0％和4.0％。接受≤4剂和>4剂巴利昔单抗治疗患者的感染发生率分别为33.3％（5/15）和80.0％（8/10）（*P*＝0.214）。在随访≥1年的16例患者中，中性粒细胞减少、贫血和血小板减少的发生率分别为6.3％、12.5％和12.5％。病毒感染、细菌感染和真菌感染的发生率分别为25.0％、25.0％和0。该治疗组未观察到新发的3～4级肝肾功能异常、细胞因子释放综合征。

非巴利昔单抗治疗组，中性粒细胞减少、贫血和血小板减少的发生率分别为25.0％、25.0％和18.6％。任何感染、病毒感染、细菌感染和真菌感染的发生率分别为37.5％、18.8％、25.0％和6.3％。

## 讨论

本研究首次报道了巴利昔单抗可有效控制GI-cGVHD，且其血液学不良反应及感染并发症发生率与我中心既往数据相当[10]。既往我中心研究证实[23]，巴利昔单抗治疗激素难治性急性GVHD的安全性良好，其总体感染率、病毒感染率、细菌感染率及真菌感染率与其他cGVHD系统免疫抑制剂（如芦可替尼、甲氨蝶呤）相当[24]。虽然本研究血液学不良反应发生率似乎高于急性GVHD相关研究[25]，但cGVHD本身就是导致移植物功能不良的重要危险因素，故骨髓抑制现象不能完全归因于巴利昔单抗。这表明巴利昔单抗治疗GI-GVHD具有可接受的安全性。

根据EBMT-NIH-CIBMTR工作组2018发布的cGVHD预后危险评分系统[26]，患者移植时高龄、合并早期aGVHD、cGVHD与移植间隔<5个月、cGVHD发生时血清胆红素≥34.2 µmol/L（2 mg/dl）、cGVHD发生时的KPS（Karnofsky）功能状态评分<80分、cGVHD发生时外周血PLT<100×10^9^/L、供者来源为其他相关/错配的无关供者（2个及以上位点不合）、移植时疾病状况为中晚期、性别错配（女供男）、GVHD预防（他克莫司+甲氨蝶呤+其他T细胞清除）、cGVHD发生时外周血淋巴细胞计数<1.0×10^9^/L、cGVHD发生时外周血嗜酸性粒细胞计数<0.5×10^9^/L均为预后不良因素。本研究25例接受巴利昔单抗治疗患者中60％预后较差。

T细胞活化在cGVHD发病机制中起关键作用。细胞毒性T细胞（CTL）通过穿孔素/颗粒酶途径诱导肠上皮细胞凋亡、Fas/FasL介导的凋亡途径激活导致特征性“隐窝凋亡”病理改变、TNF-α等细胞因子破坏肠黏膜紧密连接等被证实参与了肠道GVHD的发病。因此，阻断T细胞活化对治疗GI-cGVHD预后至关重要。本研究中接受巴利昔单抗治疗的GI-cGVHD患者均≥2级，且主要为3～4级（84％）。初始治疗的低缓解率提示，糖皮质激素联合钙调磷酸酶抑制剂对T细胞活化的抑制强度可能不足以控制GI-cGVHD，而目前对于GI-cGVHD尚无明确有效方案。

巴利昔单抗通过靶向IL-2受体抑制活化T细胞，从而发挥强效抗GVHD作用。Chakupurakal等[27]报道7例激素难治性cGVHD患者接受巴利昔单抗治疗（第1、4天各20 mg），其中5例（71％）获得PR，皮肤、口腔及肝脏症状改善，但未提供GI-cGVHD相关数据。我中心前期研究发现，巴利昔单抗治疗激素难治性急性GVHD的安全性良好，其总体感染率、病毒感染率、细菌感染率和真菌感染率分别为45.0％、35.0％、30.0％和10.0％[23],[28]，这一数据与cGVHD治疗中其他全身性免疫抑制剂（如芦可替尼和甲氨蝶呤）的感染发生率具有可比性。进一步研究提示巴利昔单抗有助于控制严重肝脏cGVHD[10]，且其血液学不良反应和感染性毒性发生率与对照组相当。本研究中，未接受巴利昔单抗治疗患者基线腹泻级别均为1～2级，其中75.0％为1级，与其相比，接受巴利昔单抗患者的基线腹泻程度更重（均≥2级，84％为3～4级）。对cGVHD一线治疗疗效进行初步评估至少需要1个月时间[2],[9]，而在此期间患者腹泻量可能已显著升高，甚至出现消化道出血、肠梗阻等严重合并症，这将极大增加后续治疗难度。因此，本研究中接受巴利昔单抗治疗的患者起始治疗时间为初始治疗启动后中位时间6（1～13）d，这与治疗严重肝脏cGVHD原则相似[10]。尽管接受巴利昔单抗治疗的患者同时接受了其他系统免疫抑制剂治疗，且这些治疗对GVHD的影响不能完全排除，但本项初步研究显示，联合巴利昔单抗治疗的CR率高达88.0％，2年OS率为75.4％，患者的OS率、2年累积复发率、2年非复发死亡率和对照组相比差异均无统计学意义，提示其确实可能具有治疗GI-cGVHD的潜力。进一步可通过前瞻性随机对照试验证实其有效性。

综上所述，本研究发现巴利昔单抗联合糖皮质激素及钙调磷酸酶抑制剂有助于有效控制GI-cGVHD，安全性在可接受范围，可能为该类患者提供新的治疗选择。但本研究样本量较少、部分病例随访时间较短，未来应开展前瞻性临床研究纳入更大样本量验证其安全性。此外，GI-cGVHD患者对药物反应的遗传学和病理生理学机制仍需要进一步探索。
